# Location of pathogenic variants in *PSEN1* impacts progression of cognitive, clinical, and neurodegenerative measures in autosomal‐dominant Alzheimer's disease

**DOI:** 10.1111/acel.13871

**Published:** 2023-06-08

**Authors:** Stephanie A. Schultz, Zahra Shirzadi, Aaron P. Schultz, Lei Liu, Colleen D. Fitzpatrick, Eric McDade, Nicolas R. Barthelemy, Alan Renton, Bianca Esposito, Nelly Joseph‐Mathurin, Carlos Cruchaga, Charles D. Chen, Alison Goate, Ricardo Francisco Allegri, Tammie L. S. Benzinger, Sarah Berman, Helena C. Chui, Anne M. Fagan, Martin R. Farlow, Nick C. Fox, Brian A. Gordon, Gregory S. Day, Neill R. Graff‐Radford, Jason J. Hassenstab, Bernard J. Hanseeuw, Anna Hofmann, Clifford R. Jack, Mathias Jucker, Celeste M. Karch, Robert A. Koeppe, Jae‐Hong Lee, Allan I. Levey, Johannes Levin, Ralph N. Martins, Hiroshi Mori, John C. Morris, James Noble, Richard J. Perrin, Pedro Rosa‐Neto, Stephen P. Salloway, Raquel Sanchez‐Valle, Peter R. Schofield, Chengjie Xiong, Keith A. Johnson, Randall J. Bateman, Reisa A. Sperling, Jasmeer P. Chhatwal, the Dominantly Inherited Alzheimer Network Investigators

**Affiliations:** ^1^ Massachusetts General Hospital, Harvard Medical School Boston Massachusetts USA; ^2^ Brigham and Women's Hospital Boston Massachusetts USA; ^3^ Ann Romney Center for Neurologic Diseases Boston Massachusetts USA; ^4^ Washington University in St. Louis School of Medicine St. Louis Missouri USA; ^5^ Department of Genetics and Genomic Sciences Icahn School of Medicine at Mount Sinai New York New York USA; ^6^ INEBA Buenos Aires Argentina; ^7^ University of Pittsburgh Pittsburgh Pennsylvania USA; ^8^ Department of Neurology, Keck School of Medicine University of Southern California Los Angeles California USA; ^9^ Indiana Alzheimer's Disease Research Center Indianapolis Indiana USA; ^10^ Dementia Research Centre & UK Dementia Research Institute UCL Institute of Neurology London UK; ^11^ Mayo Clinic Jacksonville Florida USA; ^12^ Institute of Neuroscience, UCLouvain Brussels Belgium; ^13^ Gordon Center for Medical Imaging in the Radiology Department of MGH Boston Massachusetts USA; ^14^ German Center for Neurodegenerative Diseases (DZNE) Tuebingen Germany; ^15^ Mayo Clinic Rochester Minnesota USA; ^16^ University of Michigan Ann Arbor Michigan USA; ^17^ Asan Medical Center University of Ulsan College of Medicine Seoul South Korea; ^18^ Emory Goizueta Alzheimer's Disease Research Center Atlanta Georgia USA; ^19^ German Center for Neurodegenerative Diseases (DZNE) Munich Germany; ^20^ Department of Neurology Ludwig‐Maximilians‐Universität München Munich Germany; ^21^ Munich Cluster for Systems Neurology (SyNergy) Munich Germany; ^22^ Edith Cowan University Joondalup Western Australia Australia; ^23^ Osaka City University Medical School Osaka Japan; ^24^ Columbia University New York New York USA; ^25^ Translational Neuroimaging Laboratory, Le Centre intégré universitaire de santé et de services sociaux (CIUSSS) de l'Ouest‐de‐l'Île‐de‐Montréal; Department of Neurology and Neurosurgery McGill University Montreal Canada; ^26^ Butler Hospital Providence Rhode Island USA; ^27^ Alzheimer's disease and other cognitive disorders Unit, Neurology Department, Hospital Clínic de Barcelona Institut d'Investigacions Biomediques Barcelona Spain; ^28^ Neuroscience Research Australia Randwick New South Wales Australia; ^29^ School of Medical Sciences University of New South Wales Sydney New South Wales Australia

**Keywords:** Autosomal dominant Alzheimer disease (ADAD), heterogeneity, neurodegeneration, *Presenilin‐1*, *PSEN1*

## Abstract

Although pathogenic variants in *PSEN1* leading to autosomal‐dominant Alzheimer disease (ADAD) are highly penetrant, substantial interindividual variability in the rates of cognitive decline and biomarker change are observed in ADAD. We hypothesized that this interindividual variability may be associated with the location of the pathogenic variant within *PSEN1. PSEN1* pathogenic variant carriers participating in the Dominantly Inherited Alzheimer Network (DIAN) observational study were grouped based on whether the underlying variant affects a transmembrane (TM) or cytoplasmic (CY) protein domain within PSEN1. CY and TM carriers and variant non‐carriers (NC) who completed clinical evaluation, multimodal neuroimaging, and lumbar puncture for collection of cerebrospinal fluid (CSF) as part of their participation in DIAN were included in this study. Linear mixed effects models were used to determine differences in clinical, cognitive, and biomarker measures between the NC, TM, and CY groups. While both the CY and TM groups were found to have similarly elevated Aβ compared to NC, TM carriers had greater cognitive impairment, smaller hippocampal volume, and elevated phosphorylated tau levels across the spectrum of pre‐symptomatic and symptomatic phases of disease as compared to CY, using both cross‐sectional and longitudinal data. As distinct portions of PSEN1 are differentially involved in APP processing by γ‐secretase and the generation of toxic β‐amyloid species, these results have important implications for understanding the pathobiology of ADAD and accounting for a substantial portion of the interindividual heterogeneity in ongoing ADAD clinical trials.

## Graphical abstract

Although pathogenic variants in PSEN1 leading to autosomal‐dominant Alzheimer disease (ADAD) are highly penetrant, substantial interindividual variability in the rates of cognitive decline and biomarker change are observed in ADAD. PSEN1 variant carriers participating in the Dominantly Inherited Alzheimer Network were grouped based on whether the underlying variant affects a transmembrane (TM) or cytoplasmic (CY) protein domain within PSEN1. While both CY and TM groups were found to have similarly elevated beta‐amyloid burden compared to non‐carriers, TM carriers had greater cognitive impairment, smaller hippocampal volume, and elevated phosphorylated tau levels compared to CY carriers.

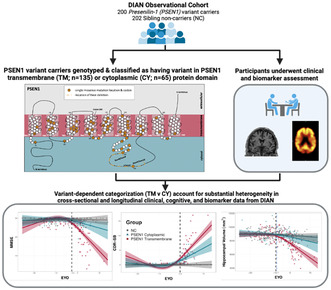

**Abbreviations**
AAO: expected age at symptom onset
ADAD: autosomal dominant Alzheimer's disease
APP: Amyloid Precursor protein
CDR‐SB: Clinical dementia Rating ‐ Sum of Boxes
CSF: Cerebrospinal fluid
CY: Cytoplasmic
DIAN‐Obs: Dominantly Inherited Alzheimer's Network ‐ Observational Study
EYO: Expected years from symptom onset
HV: Hippocampal volume
IP‐MS: immunoprecipitation mass spectrometry
LMEM: Linear mixed effects model
MMSE: Mini‐mental state examination
MPRAGE: Magnetization‐prepared rapid acquisition with gradient echo
MRI: Magnetic resonance imaging
PET: Positron emission tomography
PiB: [11C] Pittsburgh compound B
PSEN1: Presenilin 1
PSEN2: Presenilin 1
ROI: Region of interest
TM: Transmembrane

## 1 INTRODUCTION

The study of autosomal‐dominant Alzheimer's disease (ADAD) is a cornerstone for understanding AD pathobiology, underpinning most animal models of AD and providing a window into the biomarker changes that precede AD dementia (Hall & Roberson, 2012; Hsu et al., 2018). The majority of ADAD disease‐causing mutations occur in presenilin 1 (*PSEN1*), in which over 300 distinct pathogenic variations have been identified (Psen‐1|Alzforum, n.d.). Though ADAD‐causing *PSEN1* variants are highly penetrant, there is striking heterogeneity in the observed age of symptom onset (Lippa et al., 2000; Ryan et al., 2016; Ryman et al., 2014; Wegiel et al., 1998), biomarker trajectories (Klunk et al., 2007), and cognitive decline (Ryan et al., 2016; Ryan & Rossor, 2010; Tang et al., 2016) *across* individuals with different *PSEN1* variants. Particularly when used as outcome measures in clinical research, this heterogeneity in biomarker and cognitive measures presents a major challenge for ongoing clinical trials (Buckley & Knopman, 2021; Jutten et al., 2021).

AD biomarkers are playing an increasingly integral role in AD therapeutic development, as shown by the profound influence that monitoring reductions in β‐amyloid PET signal have had in recent anti‐amyloid, disease modifying therapeutic trials for AD. However, reductions in β‐amyloid burden are not necessarily accompanied by corresponding improvements in rates of cognitive and functional decline (Doody et al., 2013; Henley et al., 2019; Honig et al., 2018; Salloway et al., 2021; Sevigny et al., 2016). Accounting for the inter‐individual variability in rates of cognitive and neurodegenerative progression could improve the detection of drug effects in clinical trials. This is especially true in clinical trials with relatively small sample sizes, including ADAD clinical trials (Bateman et al., 2017; Salloway et al., 2021).

There is an increasing understanding of the phenotypic diversity of ADAD clinical and pathophysiological presentations associated with mutations in amyloid precursor protein (*APP*), *PSEN1*, and *PSEN2*. Most previous studies (Chhatwal et al., 2022; Larner, 2013; Mann et al., 2001; Pavisic et al., 2020; Ringman et al., 2014; Ryan et al., 2016; Ryan & Rossor, 2010; Shea et al., 2016; Willumsen et al., 2021) have implemented broad genotype categories to investigate heterogeneity in ADAD, commonly focusing on differences between *PSEN1*, *PSEN2*, and *APP* variants or separating *PSEN1* variants based on whether the pathogenic variant occurs prior to codon 200. More recently, our group applied a more granular approach to categorizing *APP*, *PSEN1*, and *PSEN2* pathogenic variant carriers based on individual protein domains affected. While this approach accounted for substantial inter‐individual heterogeneity in β‐amyloid PET, this categorization was not predictive of individual rates of clinical progression. In addition, the granularity of this categorization presents practical challenges in terms of implementation in clinical trials with relatively small sample sizes. In this context, we examine a simple, alternative approach to categorizing the many *PSEN1* variants in the Dominantly Inherited Alzheimer's Network observational study (DIAN‐Obs) in a manner that accounts for significant heterogeneity in ADAD progression while also providing a tool that can be used to improve the design and analysis of ADAD clinical trial data.

PSEN‐1/2 forms the catalytic core of the γ‐secretase complex and consistent with the complexity of γ‐secretase function and its unusual intramembrane proteolytic activity, different domains within PSEN‐1/2 likely play unique roles in the endopeptidase activity of γ‐secretase, docking of APP and other substrates, and in determining the efficiency of processive γ‐cleavage of Aβ peptides. Furthermore, the within membrane cleavage of APP by γ‐secretase (endopeptidase activity) is critical for γ‐secretase function, and the nature of this enzymatic activity requires the direct participation of transmembrane (TM) domains of PSEN1. In addition, the cytoplasmic portions (CY) of PSEN1, broadly speaking, are important for recruiting APP substrate into the γ‐secretase complex, and potentially also for retaining the APP substrate in the γ‐secretase complex to allow for successive γ‐cleavage of APP (processivity). Therefore, we hypothesized that ADAD may progress differently in PSEN1 TM versus CY pathogenic variant carriers. We test this hypothesis using clinical, cognitive, and biomarker data from DIAN‐Obs.

## 2 METHODS

### 2.1 Participants

**FIGURE 1:**
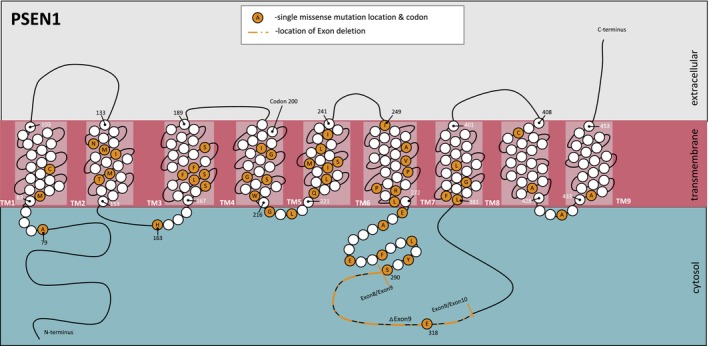
Location of included pathogenic *PSEN1* variants included in this study.

DIAN‐Obs enrolls individuals from families carrying a pathogenic variant in *PSEN1*, *PSEN2*, or *APP* leading to ADAD. We included individuals carrying *PSEN1* pathogenic variants who had completed β‐amyloid positron emission tomography (PET), magnetic resonance imaging (MRI), clinical and cognitive (Clinical Dementia Rating® SumBox (Morris, 1993) [CDR®‐SB] and Mini‐Mental State Examination (Folstein et al., 1975) [MMSE], respectively) assessment as part of their participation in DIAN‐Obs (Figure S1). Individuals were grouped based on location of the affected protein domain, namely transmembrane (TM; *N* = 135) or cytoplasmic (CY; *N* = 65) domains, using annotation available in UniProt (The UniProt Consortium et al., 2021; Figure 1). DIAN‐Obs sibling non‐carriers (NC) were included as a control group (*N* = 202). CY and TM pathogenic variant carriers (*N* = 119; mean [SD] follow‐up time interval = 3.2 [2.1] years) with available data at baseline and at least one follow‐up visit were included in longitudinal analyses.

### 2.2 Imaging analyses

#### 2.2.1 MRI and Aβ PET

MRI and PET data acquisition and processing have been described in detail in previous studies (Bateman et al., 2012; Benzinger et al., 2013). DIAN‐Obs imaging data were screened for protocol compliance and artifacts. All sites used a 3T scanner that was qualified for use at study initiation and was required to pass regular quality control assessments. Accelerated magnetization‐prepared rapid acquisition with gradient echo (MPRAGE) was acquired with repetition time/ echo time  = 2300/52.95 ms and resolution = 1.0 × 1.0 × 1.2 mm^3^. Volumetric T1‐weighted images were processed using FreeSurfer 5.3 (Fischl, 2012; Fischl et al., 2004) and the Desikan‐Killany atlas to produce regional estimates of grey matter volume within brain regions. Our primary volumetric analyses focused on the hippocampus (HV) as the a priori region of interest (ROI). In addition to HV, used for primary analyses, regional exploratory analyses examined the remaining cortical and subcortical regions (see Figure 3) for which FreeSurfer data were available. Volumetric measures were averaged across left and right hemispheres and adjusted for total intracranial volume prior to statistical analysis.

PET imaging was performed after a bolus injection of [^11^C] Pittsburgh compound B (PiB). β‐Amyloid PET acquisition consisted of a 70‐min scan starting at injection or a 30‐min scan beginning 40 min postinjection. Data in the 40–70 min postinjection window were converted to regional standardized uptake value ratios (SUVRs) relative to the cerebellar grey matter using FreeSurfer‐derived ROIs (PET Unified Pipeline, https://github.com/ysu001/PUP). Partial volume correction using a regional spread function technique was employed (Su et al., 2015). Scanner‐specific spatial filters were applied to achieve a common resolution (8 mm) across PET scanners. A composite SUVR for mean cortical Aβ deposition measure was generated using the average across the left and right lateral orbitofrontal, medial orbitofrontal, rostral middle frontal, superior frontal, superior temporal, middle temporal, and precuneus regions (Su et al., 2013, 2016). A composite partial volume‐corrected SUVRs for mean cortical Aβ deposition measure was generated.

### 2.3 CSF analyses

Cerebrospinal fluid (CSF) was obtained using procedures consistent with the biofluid protocol of the Alzheimer's Disease Neuroimaging Initiative. CSF assays for Aβ40, and Aβ42 and phospho‐tau 181 were performed using an automated immunoassay system (LUMIPULSE G1200; Fujirebio). CSF samples were additionally analyzed by nano liquid chromatography coupled to high‐resolution tandem mass spectrometry using parallel reaction monitoring and higher energy C‐trap dissociation fragmentation as previously described (Barthélemy et al., 2020). Further detail can be found in Supplementary Methods.

### 2.4 Statistical analyses

Primary analyses examined potential cross‐sectional differences in clinical and core biomarker measures between the NC, TM, and CY groups. To evaluate group differences in clinical and cognitive functioning, HV volume, Aβ burden, and phospho‐tau levels across the disease course, we used a series of multi‐variate linear mixed effects models (LMEM; lme4 package (Bates et al., 2015) in R; see Supplementary Methods for more details). LMEMs included years of education (for clinical and cognitive outcomes), age at visit, sex, *APOE ε*4 status, expected years from symptom onset (EYO), Group (NC, CY, or TM), and an EYO by Group interaction as fixed effects. As done in previous studies in this cohort (Gordon et al., 2018; Mishra et al., 2018; Preische et al., 2019), to improve model fit for CDR‐SB, MMSE, and MRI outcome measures, EYO was modeled as a restricted cubic spline with knots at the 0.10, 0.50, and 0.90 quantiles to allow for assessment of non‐linear effects. A random effect for family membership was included to account for shared variance that may exist among family members. The linear or cubic EYO by Group interaction between the CY and TM groups is the main term of interest and test statistics for this term are reported in the main results. All other between‐group comparisons (TM vs. CY, TM vs. NC, and CY vs. NC) are reported in Table 2. The Benjamini–Hochberg method was applied to CSF analyses to account for multiple comparisons.

## 3 RESULTS

### 3.1 Description of the cohort

Baseline demographic characteristics are reported in Table 1. The CY group was younger (mean [SD] age = 34.6 [10.0] years old) compared to the TM group (mean [SD] age = 38.5 [10.6] years old), had a higher percentage of APOE *ε*4 carriers, and earlier mean EYO (see Supplementary Methods for calculation of EYO). EYO, chronological age, and *APOE ε*4 carrier status were therefore included as covariates in primary analyses. Notably, chronological age and EYO measures were statistically similar between the CY and TM groups in the subset of individuals with available longitudinal data (Table S2).

**TABLE 1:** Cross‐sectional background characteristics.

Characteristic	NC *N* = 202	CY *N* = 65	TM *N* = 135
Female, %	58.5	56.5	62.4
Education, years	15.0 (2.8)	14.5 (2.8)	14.6 (3.2)
*APOE* ε4 +, %	30.1	41.9	24.0^b^
Age at visit, years	37.4 (11.2)	34.6 (10.0)^a^	38.5 (10.6)^b^
AAO, years	48.8 (6.3)	46.7 (7.2)	46.4 (7.8)
EYO, years	−10.6 (11.7)	−10.5 (11.7)	−6.8 (10.3)^a^ ^,^ ^b^

*Note*: Mean (SD) presented unless otherwise specified. Chi‐square and *t* tests evaluated between‐group differences on background characteristics. Characteristics identified as significantly different between groups were included as covariates in primary analyses.

Abbreviations: AAO, expected age at symptom onset; CY, cytoplasmic; EYO, expected years to symptom onset; TM, transmembrane.

^a^ Indicates significant difference from the NC group (*p* < 0.05).

^b^ Indicates significant difference from the CY group (*p* < 0.05).

#### 3.1.1 Baseline clinical and cognitive measures vary between the TM and CY groups across EYO

**FIGURE 2:**
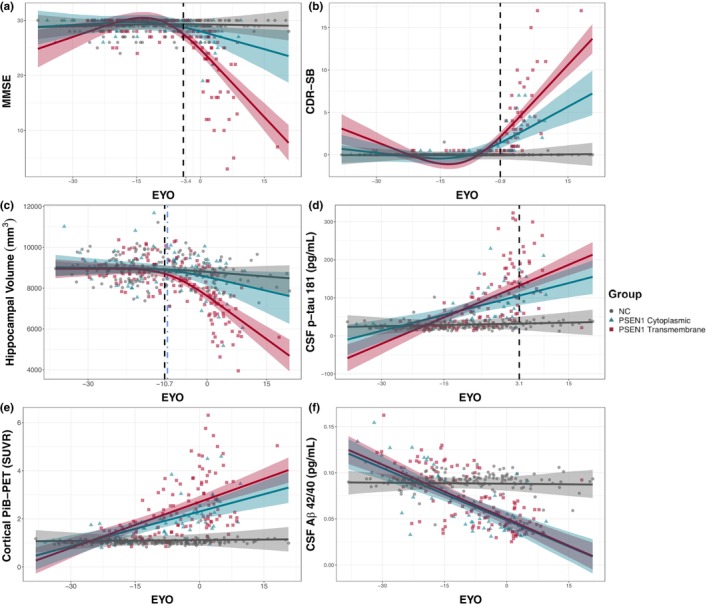
CY and TM groups differ on clinical, cognitive, and neurodegenerative measures, but not on measures of AD molecular pathology. Cross‐sectional (a) Mini‐Mental State Examination (MMSE), (b) Clinical Dementia Rating‐SumBox (CDR‐SB), (c) hippocampal volume (mm^3^), (d) CSF phospho‐tau 181 (pg/mL), (e) Composite PiB‐PET (SUVR), and (f) CSF Aβ 42/40 (pg/mL) values for non‐carriers (NC; grey circles), *PSEN1* Cytoplasmic (CY) pathogenic variant carriers (blue triangles), and *PSEN1* transmembrane (TM) pathogenic variant carriers (red squares), as compared to expected years to symptom onset (EYO). The solid line represents the median value of model estimates and the shaded areas represent the 99% credible intervals around the model estimates derived by the Hamiltonian Markov chain Monte Carlo analyses. The black dotted lines in panels a–e indicate the first EYO where the TM and CY groups began to significantly diverge on cross‐sectional measures and was determined to the first point where the 99% credible intervals around the difference distribution between the TM and CY groups did not overlap 0 (See Figure S2). This corresponds to an EYO of −3.8 for MMSE (a), an EYO of −1.1 for CDR‐SB (b), an EYO of −10.9 for hippocampal volume (c), an EYO of 3.1 for CSF phospho‐tau181 (d), and EYO of −8.4 for PiB‐PET (e). The blue dot‐dashed line indicates the point of divergence between the CY and TM group curves on cross‐sectional hippocampal volume after accounting for concurrent levels of PiB‐PET and CSF phospho‐tau 181 in addition to demographic covariates (see section 2 and Supplementary Methods for additional details). Note that the point of divergence in hippocampal volume is largely unchanged after adjusting for PiB‐thePET signal and CSF phospho‐tau 181 levels. Each outcome measure was first modeled using both cubic and linear terms for EYO (see section 2, Supplementary Methods, and Table 2). Cubic fit terms were retained if they significantly improved model fit (as in panels a–c) and dropped (as in panels d–f) if they did not significantly improve model fit for each cognitive or biomarker measures (Table 2).

Both TM and CY groups demonstrated significantly lower MMSE scores with increasing EYO compared to the NC group (Table 2). In addition, the TM group had lower MMSE scores with increasing EYO as compared to the CY group (cubic EYO*Group: B [SE] = −0.51 [0.11] and *p* = 1.23e‐06; Table 2). Divergence analyses revealed TM and CY groups began to diverge on MMSE starting at an EYO = −3.8 years (Figure 2a and Figure S2A) with more rapid MMSE decline in the TM group.

**TABLE 2:** Differences between the PSEN1 cytoplasmic (CY) carrier, transmembrane (TM) carrier, and non‐carrier (NC) groups on neurodegeneration, amyloid, and clinical outcomes across the disease.

Model outcome	Model term	TM vs. NC	CY vs. NC	TM vs. CY
MMSE	Linear EYO* group	B [SE] = −0.29 [0.07] *p* = 2.86e‐05	ns	B [SE] = −0.24 [0.08] *p* = 0.005
	Cubic EYO* group	B [SE] = −0.70 [0.07] *p* < 2e‐16	ns	B [SE] = −0.51 [0.11] *p* = 1.23e‐06
CDR‐SB	Linear EYO* group	B [SE] = 0.21 [0.04] *p* = 1.06e‐08	ns	B [SE] =0.15 [0.05] *p* = 7.66e‐04
	Cubic EYO* group	B [SE] = 0.46 [0.04] *p* < 2e‐16	B [SE] = 0.21 [0.05] *p* = 1.07e‐05	B [SE] = 0.26 [0.06] *p* = 2.44e‐06
HV	Linear EYO* group	B [SE] = −50.69 [20.1] *p* = 0.012	ns	ns
	Cubic EYO* group	B [SE] = −138.19 [22.19] *p* = 1.33e‐09	ns	B [SE] = −86.2 [31.7] *p* = 0.007
PiB‐PET	Linear EYO* group	B [SE] = 0.0 [0.01] *p* < 2e‐16	B [SE] = 0.05 [0.01] *p* = 3.26e‐10	ns

*Note*: Unstandardized beta‐weights (B), standard errors (SE), and *p* values for between group comparisons (TM vs. NC; CY vs. NC; TM vs. CY) for estimated years to symptom onset (EYO) by group model terms for outcomes of interest. Each outcome measure was first modeled using both cubic and linear terms for EYO. Cubic fit terms were retained if they significantly improved model fit (MMSE, CDR‐SB, and HV) and dropped (PiB‐PET) if they did not significantly improve model fit for each cognitive or biomarker measures. See section 2 and Supplementary Methods for additional details about the models. Comparisons with *p* > 0.05 are listed as not significant (ns).

Similar to results with MMSE, the TM and CY groups exhibited significantly greater CDR‐SB with increasing EYO compared to NC (Table 2). In addition, the TM group had significantly greater CDR‐SB with increasing EYO as compared to the CY group (cubic EYO*Group: B [SE] = 0.26 [0.06] and *p* = 2.44e‐06; Table 2). Divergence analyses revealed TM and CY groups began to diverge on CDR‐SB score starting at an EYO = ‐0.9 years (Figure 2b and Figure S2B), with significantly greater increases in CDR‐SB in the TM group.

#### 3.1.2 Baseline regional brain volumes vary between the TM and CY groups

We next examined whether differences in brain atrophy were present across the CY and TM groups using HV. We observed that the TM group had significantly lower HV with respect to EYO compared to the NC group (Table 2) and CY group (cubic EYO*Group: B [SE] = −86.22 [31.66] and *p* = 0.007), suggesting greater HV loss for a given EYO in TM carriers compared to CY carriers, particularly as carriers approached their familial age of symptom onset. Divergence analyses revealed HV significantly diverged between the TM and CY groups starting at an EYO of −10.9 years (Figure 2c and Figure S2C) with greater HV loss in the TM group.

**FIGURE 3:**
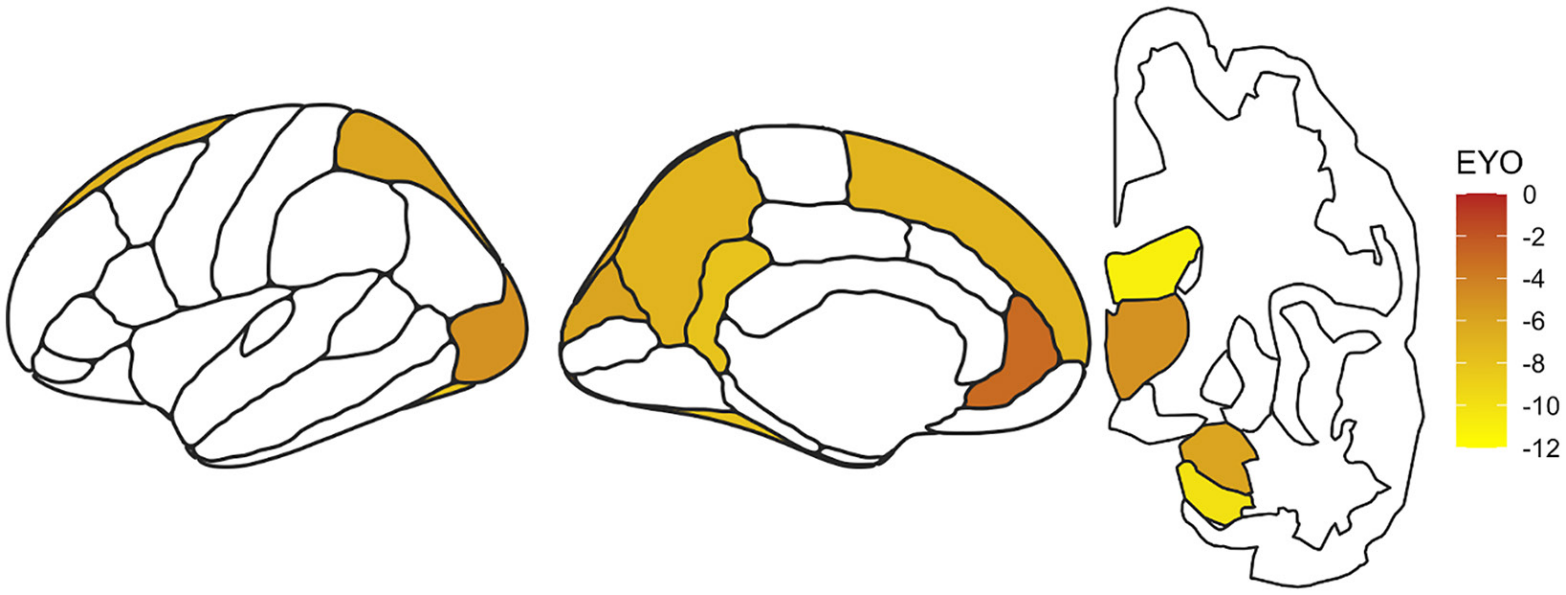
Hippocampus and several midline cortical and sub‐cortical regions show greater neurodegeneration in TM carriers as compared to CY carriers. Using FreeSurfer‐defined cortical and sub‐cortical regions of interest, we compared volumetric measures between the *PSEN1* cytoplasmic (CY) and transmembrane (TM) groups across the disease continuum. A number of midline cortical and subcortical regions showed greater volume loss with respect to EYO in the TM group versus the CY group (colored regions). Colors depict the EYO at which divergence between the TM and CY groups was observed. EYO = expected years to symptom onset.

To examine the broader anatomy of neurodegenerative differences between the TM and CY groups, we also conducted exploratory volumetric analyses across a set of FreeSurfer‐defined brain regions. The TM group had significantly smaller volumes in many cortical regions including the superior frontal, rostral anterior cingulate, isthmus cingulate, precuneus, cuneus, superior parietal, lateral occipital, fusiform, amygdala, and putamen compared to the CY group. For the majority of these regions, divergence between the CY and TM groups was observed at an EYO of approximately −11 years (Figure 3). Divergence between the CY and TM groups in lateral ventricle volume (used here as a measure of central atrophy) was similarly observed at an EYO of −10.9 years.

#### 3.1.3 Differences in neurodegeneration across the CY and TM groups account for variations in cognition

As neurodegenerative and cognitive measures may be linked, we assessed whether group‐based differences in HV account for group differences in cognition. Group differences in HV fully mediated group differences on MMSE scores. Specifically, the direct effect (*β* = −0.57, *p* = 0.004) of *PSEN1* grouping on MMSE was not significant when HV was included as a mediator (mediator effect was *β* = 0.67, *p* < 2e‐16; residual direct effect was *β* = −0.12, *p* = 0.460; Figure S3).

#### 3.1.4 Differences in β‐amyloid and phospho‐tau across the CY and TM groups

Next, we assessed cross‐sectional differences between the TM and CY groups in several available measures of Aβ and CSF phospho‐tau. The CY and TM groups did not significantly differ on a cortical composite PiB‐PET measure across EYO (linear EYO*Group: B [SE] = 0.02 [0.01] and *p* = 0.056; Figure 2e; Table 2). Further exploration of group differences in the precuneus, one of the earliest regions in ADAD to accumulate amyloid (Benzinger et al., 2013), revealed there was also no significant difference between the TM and CY groups in regional amyloid burden across EYO (linear EYO*Group: B [SE] = 0.01 [0.01] and *p* = 0.059). Additionally, we examined immunoassay‐based measures of CSF Aβ 42/40 ratio and observed no significant differences between the CY and TM groups (Table S3). This indicates that despite differences across the CY and TM groups with respect to CDR‐SB, MMSE, and neurodegenerative measures, no clear group differences were present in these commonly‐used measures of β‐amyloid burden.

In a subset of individuals with available data (*N* = 225), we next examined a series of immunoprecipitation mass spectrometry (IP‐MS) based measures of tau phosphorylated at residues 181, 202, 205, and 217. Prior work indicates that these phospho‐tau species change at different points in the course of the disease. As in prior studies (Barthélemy et al., 2020), ratios of phosphorylated residues to non‐phosphorylated residues were used as the primary measure of tau phosphorylation at a specific site. While IP‐MS pT217/T217 was significantly different between CY and TM across EYO (B [SE] = 0.09 [0.03] and *p* = 0.018), all other examined IP‐MS CSF phospho‐tau proteoforms were statistically similar between the CY and TM groups (Table S3; Figure S4). The CY and TM groups were significantly different on immunoassay‐based measures of phospho‐tau 181 (B [SE] = 1.8 [0.6] and *p* = 0.006; Table S3; Figure 2d).

We performed an additional sensitivity analysis to examine whether controlling for these core measures of AD pathology impacted the relationship between PSEN1 grouping and HV. Terms for β‐amyloid (PiB‐PET composite SUVR) and phospho‐tau (CSF Lumipulse phospho‐tau 181) were included as fixed effects in LMEMs assessing the effects of group membership on HV. Results remained unchanged. Divergence analyses revealed HV diverged between the TM and CY groups starting at an EYO of −10.1 (Figure 2c) after these additional measures of AD pathology were included in the model as covariates.

#### 3.1.5 Alternative variant grouping does not account for neurodegenerative, clinical, or cognitive heterogeneity

Several studies, have investigated the association between biomarkers and ADAD genotype by grouping *PSEN1* pathogenic variant carriers based on whether the pathogenic variant occurs before or after codon 200 (Chhatwal et al., 2022; Ryan et al., 2016; Tang et al., 2016). As previously described (Chhatwal et al., 2022), our group reported higher cortical and striatal Aβ burden in individuals with pre‐codon 200 *PSEN1* pathogenic variants compared to post‐codon 200. However, in this previous study, no significant differences between groups were observed in CDR‐SB or CSF Aβ42/40. Therefore, to further examine the potential utility of the TM/CY categorization compared to the pre‐/post‐codon 200 categorization, we assessed several biomarker and cognitive outcomes not evaluated in the previous report (i.e., MMSE, hippocampal volume, and CSF phosph‐tau181; term of interest: EYO*group) using the codon‐based categorization approach. We observed that individuals with variants located pre‐codon 200 did not significantly differ on any of the outcomes of interest compared to the post‐codon 200 carriers (Figure S5), suggesting that the CY‐TM categorization may have greater utility compared to the codon‐based approach for these neurodegenerative, clinical, and cognitive measures.

#### 3.1.6 Longitudinal analyses of clinical, cognitive, and biomarker measures support cross‐sectional findings

**FIGURE 4:**
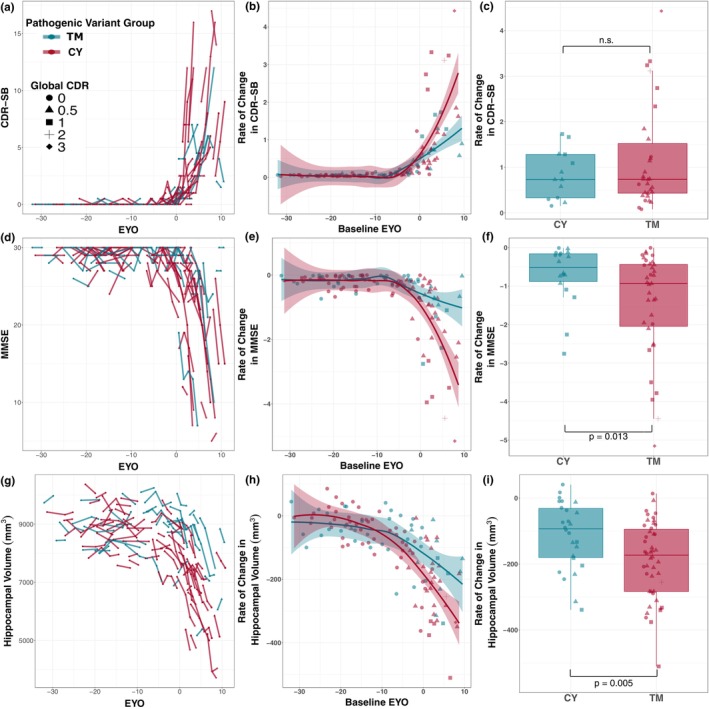
TM carriers demonstrate more rapid longitudinal cognitive decline and neurodegeneration as compared to CY carriers. Individual longitudinal trajectories, extracted annualized slopes, and group comparisons for CDR‐SB (a–c), MMSE (d–f), and HV (g–i) for TM (red) and CY pathogenic variant carriers (blue) are depicted. *t* tests were performed to compare CY versus TM annualized rates of change in CDR‐SB (c), MMSE (f), and HV (i) using the extracted slopes from individuals with a baseline EYO greater than or equal to the cross‐sectional EYO divergence point across the TM and CY groups (See Figure 2; EYO ≥ −0.9 years for CDR‐SB, EYO ≥ −3.4 years for MMSE, and EYO ≥ −10.7 years for HV). CDR‐SB, Clinical dementia rating‐SumBox score; CY, cytoplasmic domain; EYO, expected years to symptom onset; HV, hippocampal volume; MMSE, Mini‐Mental State Examination; TM, transmembrane domain.

Using longitudinal clinical, cognitive, and MRI data from *PSEN1* pathogenic variant carriers (TM group *N* = 75 and CY group *N* = 44; Figure 4a,d,g), we explored whether rates of change in MMSE, CDR‐SB, and HV differed between CY and TM pathogenic variant carriers (See Figure 4b,e,h). Similar to the results using cross‐sectional data, we observed that the TM group had significantly greater annualized rates of change on MMSE after baseline EYO of −3.4 years (*t* [50] = 2.57, *p* = 0.013; Figure 4f) and HV atrophy after baseline EYO of −10.7 years (*t* [57] = 2.90, *p* = 0.005; Figure 4i) compared to the CY group. The TM and CY groups were similar on rates of change in CDR‐SB (*t* [41] = 1.31, *p* = 0.196; Figure 4c), however.

## 4 DISCUSSION

In this observational study of individuals with ADAD, we investigated whether cross‐sectional and longitudinal clinical, cognitive, and biomarker trajectories differed based on the portion of PSEN1 affected by the pathogenic variant. We observed that individuals carrying a pathogenic variant affecting one of the nine transmembrane domains of PSEN1 had more rapid clinical, cognitive, and neurodegenerative progression of disease compared to those with variants affecting cytoplasmic domains. Differences in brain atrophy between TM and CY carriers were observed across a wide set of AD‐relevant brain regions, including the hippocampus. While measures of tau pathology (including phospho‐tau 181 and 217) differed between the CY and TM groups as well, though to a lesser extent, amyloid burden was not significantly different across the CY and TM groups. This suggests that differences in brain atrophy and cognitive trajectories may not be directly explained by core measures of AD pathology. Taken together, these results suggest that accounting for whether underlying pathogenic variants affect CY or TM domains may be beneficial in the design and analysis of ADAD clinical trials.

Notably, group differences in HV, lateral ventricle size, and midline cortical regions between the CY and TM groups were present 10 years or more prior to the expected age of symptom onset. Many of the regions of interest that varied between the CY and TM groups have been previously implicated in AD progression, including the precuneus, posterior cingulate, and isthmus cingulate. Observed differences between the CY and TM groups on HV were independent of concurrent levels of Aβ PET or CSF phospho‐tau 181. In line with these results, past studies have identified TM pathogenic variants with impairments in intracellular calcium regulation (Psen‐1|Alzforum, n.d.), raising the possibility that dysregulation of calcium homeostasis may contribute to downstream neurodegeneration independent of Aβ and tau. Additionally, the γ‐secretase complex is involved in the processing of many protein substrates beyond APP (De Strooper et al., 1998; Wolfe et al., 1999), and it remains possible that dysregulation of non‐APP‐related signaling pathways (e.g., NOTCH signaling) may partially account for the effects seen here. The wide anatomical distribution of these gray matter volume effects observed across the PSEN1 groups suggest that TM variants as a group may alter APP processing in a manner that is fundamentally more neurotoxic as compared to CY variants.

With respect to clinical and cognitive impairment, we observed that on CDR‐SB in both the CY and TM groups began to diverge from NC at a similar EYO and that the CY and TM groups had similar age of familial symptom onset. However, the findings here indicate that, compared to CY carriers, TM carriers may have more rapid decline in cognitive performance and functional status once the decline phase of ADAD has begun. Consistent with prior literature that suggests brain tissue loss is a proximal cause of cognitive decline and functional impairment (Jack et al., 1992; Risacher et al., 2010), the TM and CY group differences on clinical and cognitive measures were fully statistically mediated by group differences in HV change across EYO. Longitudinal analyses of HV, CDR‐SB, and MMSE largely mirrored cross‐sectional findings, and demonstrated that individuals with a pathogenic variant in TM domains have greater rates of decline on cognitive and neurodegenerative measures across the EYO spectrum compared to CY carriers. These longitudinal observations further support the potential importance of considering pathogenic variant location in current and future ADAD clinical trials.

We observed no group differences between the CY and TM groups across several imaging and biofluid measures of Aβ pathologies and small differences on tau measures. Previous work examining variant‐dependent heterogeneity in CSF and PET measures of Aβ found grouping individuals with pathogenic variants in *PSEN1*, *PSEN2*, or *APP* based on the affected protein domain accounted for variability in Aβ biomarkers (Chhatwal et al., 2022). However, in this previous work, some TM groups were observed to have relatively high levels of Aβ PET signal (e.g., TM domains 3 and 5) whereas others were observed to have relatively low levels of Aβ PET signal (e.g., TM domains 6 and 8). This variation in Aβ PET within individual TM domain groupings may help explain the lack of an observed difference in Aβ PET signal between the TM and CY groups observed here, as all TM domains were grouped together. More importantly, a consistent finding here and in this prior report is that levels of Aβ burden do not consistently mirror cognitive and neurodegenerative trajectories in ADAD. It remains possible that the TM and CY group differences observed here may be underpinned by toxic but as‐yet unmeasured Aβ species. Indeed, less‐commonly studied monomeric (especially Aβ 43, 38, and 37), membrane retained (Aβ 45–49) and oligomeric forms of Aβ have been associated with AD diagnosis and progression (Devkota et al., 2021; Liu, Kwak, et al., 2021; Liu, Lauro, et al., 2021). In this context, future studies examining a broader set of Aβ species and potentially neurotoxic changes in the processing of non‐APP γ‐secretase substrates will be needed to identify the mechanisms that underlie the observed differences between the CY and TM groups.

Consideration of the study population and several methodologic limitations are important to the interpretation of the results presented. The TM and CY groups differed on *APOE ε*4 carriage and baseline EYO. Though these differences were addressed in statistical models for cross‐sectional analyses and were not observed within the subset of individuals with longitudinal data, these differences remain possible confounders. Similarly, there also may be other unknown genetic, environmental, or ascertainment differences between groups. It is also notable that a consistent *APOE ε*4 carrier state effect on disease progression has not been consistently seen in ADAD. Additionally, while we employed a broad categorization of genotypes that may be useful for clinical trials and analysis, there remains substantial variability within the TM and CY groups. Information at the level of individual pathogenic variants will be needed to better identify endophenotypes within the CY and TM groups and, more broadly, among the many known ADAD pathogenic variants. On a related note, while this initial examination of variant‐dependent heterogeneity in clinical, cognitive, and biomarker measures made use of an a priori categorization of pathogenic variants (based on whether the underlying variant affects CY or TM domains in PSEN1), future work integrating biochemical information at the individual mutation‐level will likely be needed to better elucidate the mechanisms that lead to the clinical and cognitive heterogeneity observed across the TM and CY groups.

Despite these limitations, the results here support a distinction between ADAD‐causing pathogenic variants that impact CY versus TM regions within *PSEN1*, whereby TM carriers have more rapid neurodegeneration, clinical and functional decline as compared to CY carriers. Looking forward, these results have implications both for understanding the heterogeneity in ongoing ADAD clinical trials (Rabinovici, 2021), especially those employing HV or other structural MRI measures as secondary outcome measures. More broadly, these results suggest that understanding heterogeneity across the large number of ADAD causing pathogenic variants may be important both to the success of ADAD clinical trials and, more fundamentally, to our understanding of AD pathobiology.

## AUTHOR CONTRIBUTIONS

Literature search: SAS, LL, JPC; Study design: SAS, ZS, APS, LL, EM, KAJ, RJB, RAS, JPC; Data collection: SAS, JPC; Data interpretation: SAS, ZS, EM, LL, JPC; Figures: SAS, JPC; Manuscript writing: SAS, JPC; Manuscript critical review: SAS, ZS, APS, LL, CDF, EM, NRB, AR, BE, NJM, CC, CDC, AG, RFA, TLSB, SB, HCC, AMF, MRF, NCF, BAG, GSD, NRGR, JJH, BJH, AH, CRJ, MJ, CMK, RAK, JHL, AIL, JL, RNM, HM, JCM, JN, RJP, PRN, SPS, RSV, PRS, CX, KAJ, RJB, RAS, JPC.

## CONFLICT OF INTEREST STATEMENT

JPC has served on medical advisory boards for Otsuka Pharmaceuticals and Humana Healthcare. There are no conflicts.

APS has served on medical advisory boards for Janssen Pharmaceuticals and Biogen. There are no conflicts.

SMS reports consulting to Eisai, Novartis, Genentech, F. Hoffmann‐La Roche, Ltd, Gemvax, Avid Radiopharmaceuticals and Eli Lilly and Company. He also serves on the steering committees for major biomarker and clinical trials and consortia such as ADNI, DIAN, ACTC, GAP‐NET and LEADS and he is a Project Arm Leader for the DIAN‐TU study. There are no conflicts.

RJB has equity ownership interest in C2N Diagnostics and receives royalty income based on technology (stable isotope labeling kinetics and blood plasma assay) licensed by Washington University to C2N Diagnostics. He receives income from C2N Diagnostics for serving on the scientific advisory board. Washington University, with RJB as co‐inventor, has submitted the US nonprovisional patent application “Cerebrospinal fluid (CSF) tau rate of phosphorylation measurement to define stages of Alzheimer's disease and monitor brain kinases/phosphatases activity.” He has received honoraria from Janssen and Pfizer as a speaker, and from Merck and Pfizer as an advisory board member. He has been an invited speaker, advisory board member, and consultant for F. Hoffman La Roche, Ltd., an invited speaker and consultant for AC Immune and Janssen, and a consultant for Amgen and Eisai. There are no conflicts.

AMG has consulted for Eisai, Biogen, Pfizer, AbbVie, Cognition Therapeutics, and GSK. She also served on the Scientific Advisory Board of Denali Therapeutics (2015–2018). There are no conflicts.

RAS and KAJ are involved in public‐private partnership clinical trials sponsored by the NIH and Eli Lilly and Co., who owns the distribution rights to Flortaucipir (AV‐1451), but they do not have any personal financial relationship with Eli Lilly. There are no conflicts.

NGR reports grants from Biogen, grants from Abbvie, grants from Lilly outside the submitted work.

## Supporting information


Data S1

## Data Availability

Biomarker and cognitive data from the DIAN Observational Study is available by request at https://dian.wustl.edu/our‐research/observational‐study/dian‐observational‐study‐investigator‐resources/data‐request‐terms‐and‐instructions/.
