# Supplementary material for: Location of pathogenic variants in PSEN1 impacts progression of cognitive, clinical, and neurodegenerative measures in autosomal‐dominant Alzheimer's disease
Source: Aging Cell. 2023 Jun 8;22(8):e13871. doi: 10.1111/acel.13871 (PMC10410059; doi:10.1111/acel.13871)
Supplement: Supplementary file 1 — Data S1 [file ACEL-22-e13871-s001.docx]

**Supplementary Methods and Data**

**Participants:**

The DIAN-Obs enrolls families with individuals carrying pathogenic variants in *PSEN1*, *PSEN2* or *APP.* DIAN-Obs participants undergo genetic testing and repeated clinical, cognitive, fluid and brain imaging assessments ^1^. Consistent with prior literature ^2^, the large majority of ADAD families in DIAN-Obs carry pathogenic variants in *PSEN1*. Table S1 reports unique *PSEN1* variants included in this study. Individuals with pathogenic variants in intron [N = 1] and lumenal [N = 10] domain regions of *PSEN1* were excluded from these analyses due to small sample size. The majority of participants in DIAN-Obs (69% of all pathogenic variant carriers, and 93% of all *PSEN1* carriers) were included in either the CY or TM grouping. For the current study we leveraged this large population of individuals carrying a *PSEN1* mutation (DIAN-Obs data freeze version 15; last data from June 30, 2020) who had completed Aβ PET imaging, an MRI, and baseline cognitive assessment. Participants provided written informed consent or assent with proxy consent. The institutional review boards for each of the participating DIAN sites approved all aspects of the study. This study follows the Strengthening the Reporting of Observational Studies in Epidemiology ([STROBE](http://www.equator-network.org/reporting-guidelines/strobe/)) reporting guideline.

**Clinical Evaluation:**

Each DIAN participant’s EYO was calculated based on the participant's age subtracted from the expected age of symptom onset in the participant’s family. As previously described, the familial age of onset was determined through a combination of structured interviews to determine the age at onset of progressive cognitive decline for the participant’s first degree relative(s) and, if available, prior literature on the age of symptom onset for the participant’s pathogenic variant ^3^. Clinical evaluators were blind to the mutation status of participants. Clinical dementia rating (CDR) Sum of Boxes (SB) and Mini-Mental State Examination (MMSE) scores were measured for each participant using structured interviews, as previously described ^4^.

**Genotyping:**

The presence of ADAD pathogenic variants (Sanger sequencing) and *APOE* $\varepsilon$4 genotype (PCR-based) were assessed using DNA derived in parallel at the DIAN Genetics Core (DGC; Mount Sinai School of Medicine) and the National Cell Repository for Alzheimer’s Disease (NCRAD), as previously described ^5^. Concordant results between DGC and NCRAD were required for inclusion in the present dataset. Pathogenic genetic variants represented within each grouping are shown in Table S1.

**CSF Analyses:**

CSF was drawn using 21-22g Sprotte or Quincke spinal needles into polypropylene tubes, followed by placement on dry ice and shipment to the DIAN Biomarker Core at Washington University. Frozen samples were then thawed, aliquoted, and stored at -84 degrees C until assayed. CSF assays for Aβ 40, and Aβ 42 and phospho-tau 181 were performed at the DIAN Biomarker Core using an automated immunoassay system (LUMIPULSE G1200, Fujirebio, Malvern, PA) according to manufacturer's specifications.

As previously described ^6^, thawed CSF samples were additionally analyzed by nano liquid chromatography coupled to high-resolution tandem mass spectrometry (HRMS/MS) using parallel reaction monitoring and HCD fragmentation. Ratios of phosphorylation on T181, S202, T205 and T217 were measured using the ratio of the HRMS/MS transitions from phosphorylated peptides and the corresponding non-phosphorylated peptides. Each phosphorylated/non-phosphorylated peptide endogenous ratio was normalized using the ratio measured on the HRMS/MS transitions of the corresponding phosphorylated/non-phosphorylated peptide internal standards.

**Statistical Analyses:**

Primary analyses aimed to determine cross-sectional differences in clinical and pathophysiological features between the NC, TM, and CY groups across a broad range of EYO. To compare group differences on cross-sectional and longitudinal cohort background characteristics, t-tests and chi-square tests were performed, as appropriate.

*Exploratory ROI Analyses*

As a follow-up to our analysis of cross-sectional HV, we assessed broader differences in volumetric measures among CY, TM, and NC in exploratory regional analyses (summarized in Figure 3). Similar to primary analyses, multi-variate LMEMs were utilized. Separate models using each of the 42 volumetric ROIs as dependent measures were examined. Between-group divergence analyses at the level of each ROI were performed by estimating the earliest baseline EYO where significant between-group differences (CY vs. TM) were detected.

*Divergence Analyses*

Adopting the approach of prior studies from DIAN ^7–9^, selected models were examined for divergence in disease trajectories across groups using model parameters estimated from a Hamiltonian Markov Chain Monte Carlo analysis approach implemented in Stan ([http://mc-stan.org](http://mc-stan.org/)). This approach allowed the estimation of the median and 99% credible intervals for each the model fit at every baseline EYO for the NC, CY, and TM groups, as well as estimates of the distribution of the differences between groups within the 99% credible interval. Based on this difference curve and its credible interval comparing CY and TM groups, we then estimated the earliest baseline EYO at which the 99% credible intervals around the relevant group difference (e.g., TM vs. CY groups) distributions did not overlap.

*Mediation Analysis*

Mediation analysis was performed to examine whether CY vs TM group differences on cognitive functioning were statistically mediated by group differences in HV. This analysis was restricted to *PSEN1* carriers who were at an EYO > -11.0 years, based on results from primary analyses showing divergence of volumetric measures between CY and TM groups starting at around an EYO of -11 years. R (“mediation” package) was used to test for a statistical mediation. Group (CY or TM) was assessed as the independent variable, MMSE as the dependent variable, and HV as the mediator in this mediation model (Figure S3).

*Longitudinal Analyses*

A subset of 119 pathogenic variant carriers who had two or more clinical, cognitive, or biomarker assessments were used in longitudinal analyses (Figure 4A, D, and G). We leveraged data from this subset of participants to determine whether the group differences between the CY and TM groups seen in cross-sectional imaging and clinical data were also observable during longitudinal follow-up. Annualized slopes were extracted from longitudinal LMEMs where the dependent term for each model was the longitudinal measure of CDR-SB, MMSE, or HV with fixed effects terms for time from baseline. In addition to modeling a participant level random effect, both a random slope and intercept were included as random effects. For visualization purposes, individual level slopes were extracted from LMEMs and plotted across the entire EYO range (Figure 4B, E, and H). T-tests were performed to compare CY versus TM annualized rates of change in CDR-SB (Figure 4C), MMSE (Figure 4F), and HV (Figure 4I) using the extracted slopes from a subset of individuals who had a baseline EYO greater than or equal to the observed cross-sectional EYO divergence point (See Figure 2; EYO ≥ -0.9 years for CDR-SB, EYO ≥ -3.4 years for MMSE, and EYO ≥ -10.7 years for HV).

**Supplementary Methods References:**

1. Morris JC, Aisen PS, Bateman RJ, et al. Developing an international network for Alzheimer research: The Dominantly Inherited Alzheimer Network. *Clin Investig (Lond)*. 2012;2(10):975-984. doi:10.4155/cli.12.93

2. Schellenberg GD, Montine TJ. The genetics and neuropathology of Alzheimer’s disease. *Acta Neuropathol*. 2012;124(3):305-323. doi:10.1007/s00401-012-0996-2

3. Ryman DC, Acosta-Baena N, Aisen PS, et al. Symptom onset in autosomal dominant Alzheimer disease: a systematic review and meta-analysis. *Neurology*. 2014;83(3):253-260.

4. Bateman RJ, Xiong C, Benzinger TLS, et al. Clinical and Biomarker Changes in Dominantly Inherited Alzheimer’s Disease. *New England Journal of Medicine*. 2012;367(9):795-804. doi:10.1056/NEJMoa1202753

5. Bateman RJ, Xiong C, Benzinger TL, et al. Clinical and biomarker changes in dominantly inherited Alzheimer’s disease. *N Engl J Med*. 2012;367(9):795-804. doi:10.1056/NEJMoa1202753

6. Barthélemy NR, Li Y, Joseph-Mathurin N, et al. A soluble phosphorylated tau signature links tau, amyloid and the evolution of stages of dominantly inherited Alzheimer’s disease. *Nat Med*. 2020;26(3):398-407. doi:10.1038/s41591-020-0781-z

7. Preische O, Schultz SA, Apel A, et al. Serum neurofilament dynamics predicts neurodegeneration and clinical progression in presymptomatic Alzheimer’s disease. *Nat Med*. 2019;25(2):277-283. doi:10.1038/s41591-018-0304-3

8. Mishra S, Blazey TM, Holtzman DM, et al. Longitudinal brain imaging in preclinical Alzheimer disease: impact of APOE ε4 genotype. *Brain*. 2018;141(6):1828-1839. doi:10.1093/brain/awy103

9. Gordon BA, Blazey TM, Su Y, et al. Spatial patterns of neuroimaging biomarker change in individuals from families with autosomal dominant Alzheimer’s disease: a longitudinal study. *The Lancet Neurology*. 2018;17(3):241-250.

**Table S1.** List of *PSEN1* pathogenic variants included in this study.

| **Variant Protein Consequence** | **PSEN1 Grouping** |
| --- | --- |
| p.S290C;T291_S319del | CY |
| p.Ala79Val | CY |
| p.Met84Val | TM |
| p.Cys92Ser | TM |
| p.Asn135Ser | TM |
| p.Asn135Tyr | TM |
| p.Met139Val | TM |
| p.Met139Ile | TM |
| p.Ile143Thr | TM |
| p.Met146Ile | TM |
| p.Met146Leu | TM |
| p.Met146Val | TM |
| p.Thr147Ile | TM |
| p.His163Arg | CY |
| p.Ser169Leu | TM |
| p.Ser170Phe | TM |
| p.Leu171Pro | TM |
| p.Phe175del | TM |
| p.Phe176Val | TM |
| p.Ser178Pro | TM |
| p.Glu184Asp | TM |
| p.Glu184Gly | TM |
| p.Ile202Phe | TM |
| p.Gly206Ala | TM |
| p.Gly209Glu | TM |
| p.Gly209Val | TM |
| p.Gly209Ala | TM |
| p.Gly209Arg | TM |
| p.Ser212Tyr | TM |
| p.Trp215Arg | TM |
| p.Gly217Arg | CY |
| p.Gly217Val | CY |
| p.Leu219Pro | CY |
| p.Gln222His | TM |
| p.Leu226Arg | TM |
| p.Ile229Phe | TM |
| p.Ser230Asn | TM |
| p.Met233Thr | TM |
| p.Met233Leu | TM |
| p.Leu235Val | TM |
| p.Ile238Met | TM |
| p.Ile249Leu | TM |
| p.Ala260Gly | TM |
| p.Ala260Val | TM |
| p.Val261Phe | TM |
| p.Pro264Leu | TM |
| p.Pro267Leu | TM |
| p.Arg269His | TM |
| p.Leu271Val | TM |
| p.Glu273Lys | CY |
| p.Ala275Val | CY |
| p.Glu280Gly | CY |
| p.Glu280Ala | CY |
| p.Phe283Leu | CY |
| p.Leu286Val | CY |
| p.Tyr288His | CY |
| p.Ser290Cys | CY |
| p.Glu318Gly | CY |
| p.Leu381Val | TM |
| p.Gly384Ala | TM |
| p.Phe386Ser | TM |
| p.Phe386Leu | TM |
| p.Leu392Val | TM |
| p.Cys410Tyr | TM |
| p.Ala426Pro | TM |
| p.Ala431Glu | CY |
| p.Ala434Thr | TM |

**Table S2.** *Longitudinal cohort demographics and clinical characteristics.*

| **Characteristic** | **CY** | **TM** | **p-value** |
| --- | --- | --- | --- |
| **Female, %** | 59.1 | 57.9 | 0.450 |
| **Education, years** | 14.4 (2.4) | 14.4 (2.8) | 0.939 |
| **APOE** $\boldsymbol{\varepsilon}$**4 +, %** | 43.2 | 23.7 | 0.043 |
| **Baseline Age, years** | 36.2 (9.8) | 38.4 (10.5) | 0.251 |
| **AAO, years** | 46.2 (2.7) | 46.5 (7.9) | 0.875 |
| **Baseline EYO, years** | -9.2 (11.3) | -7.2 (10.3) | 0.341 |
| **Follow-up time** | 3.2 (2.1) | 3.3 (2.0) | 0.899 |

Background characteristics for subset of participants included in longitudinal analyses. Mean (SD) presented unless otherwise specified. Chi-square and t-tests evaluated between-group differences on background characteristics.

TM = Transmembrane; CY = Cytoplasmic; AAO = Expected age at symptom onset; EYO = Expected years to symptom onset.

**Table S3.** *CSF phospho-tau and Aβ measures across CY and TM groups.*

| **Biomarker** | **B (SE)** | **P-value** |
| --- | --- | --- |
| **IP-MS pT181/ T181** | **0.10 (0.08)** | **0.305** |
| **IP-MS pS202/S202** | **-0.01 (0.01)** | **0.409** |
| **IP-MS pT205/T205** | **0.007 (0.003)** | **0.076** |
| **IP-MS pT217/T217** | **0.09 (0.03)** | **0.018** |
| **LUMI ELISA pT181** | **1.8 (0.6)** | **0.006** |
| **LUMI ELISA Aβ 42/40** | **-0.001 (0.001)** | **0.409** |

Model estimates (linear EYO*Group) comparing CSF immunoprecipitation mass spectrometry (IP-MS) measures of phospho-tau isoforms, CSF immunoassay-derived (Lumipulse) phospho-tau pT181, and Aβ 42/40 across CY and TM carrier groups. P-values are adjusted for multiple comparisons using the Benjamini Hochberg method.

**Figure S1.** *Participant flowchart for cross-sectional and longitudinal samples.*

*
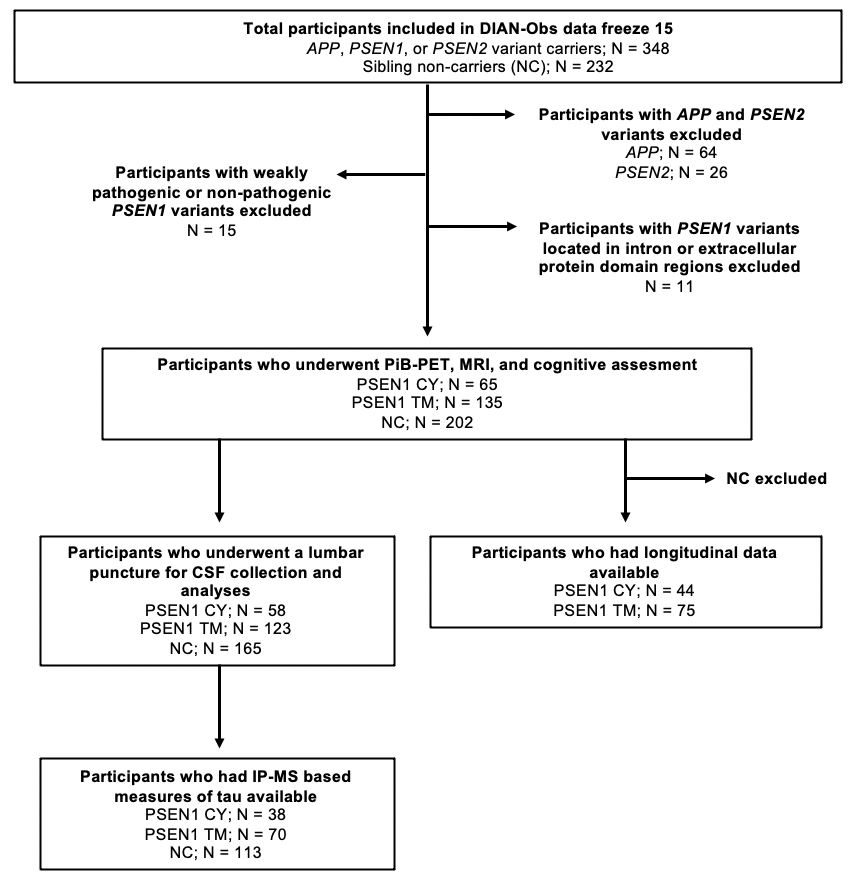
*

Flowchart shows the number of participants included in cross-sectional and longitudinal analyses.

**Figure S2. *Difference distribution curve for cross-sectional clinical, cognitive, and biomarkers in PSEN1 TM and CY pathogenic variant carriers.***

**
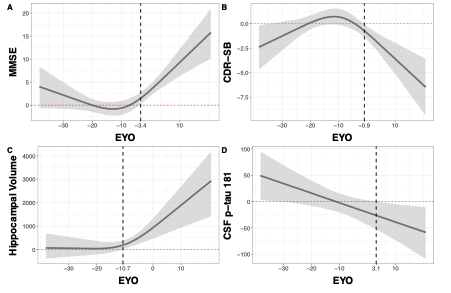
**

Difference of posterior distribution for cross-section MMSE (**A)**, CDR-SB (B), hippocampal volume (C), and CSF phosphor-tau181 (D) as a function of EYO. The solid black lines depict the median of the difference distribution; the shaded area represents the 99% equal-tailed credible intervals. EYO was considered statistically significant if the 99% equal-tailed credible intervals of the posterior distribution did not overlap 0.

**Figure S3.** *Group differences in hippocampal volume statistically mediate the effects of CY/TM grouping on cognitive performance.*

*
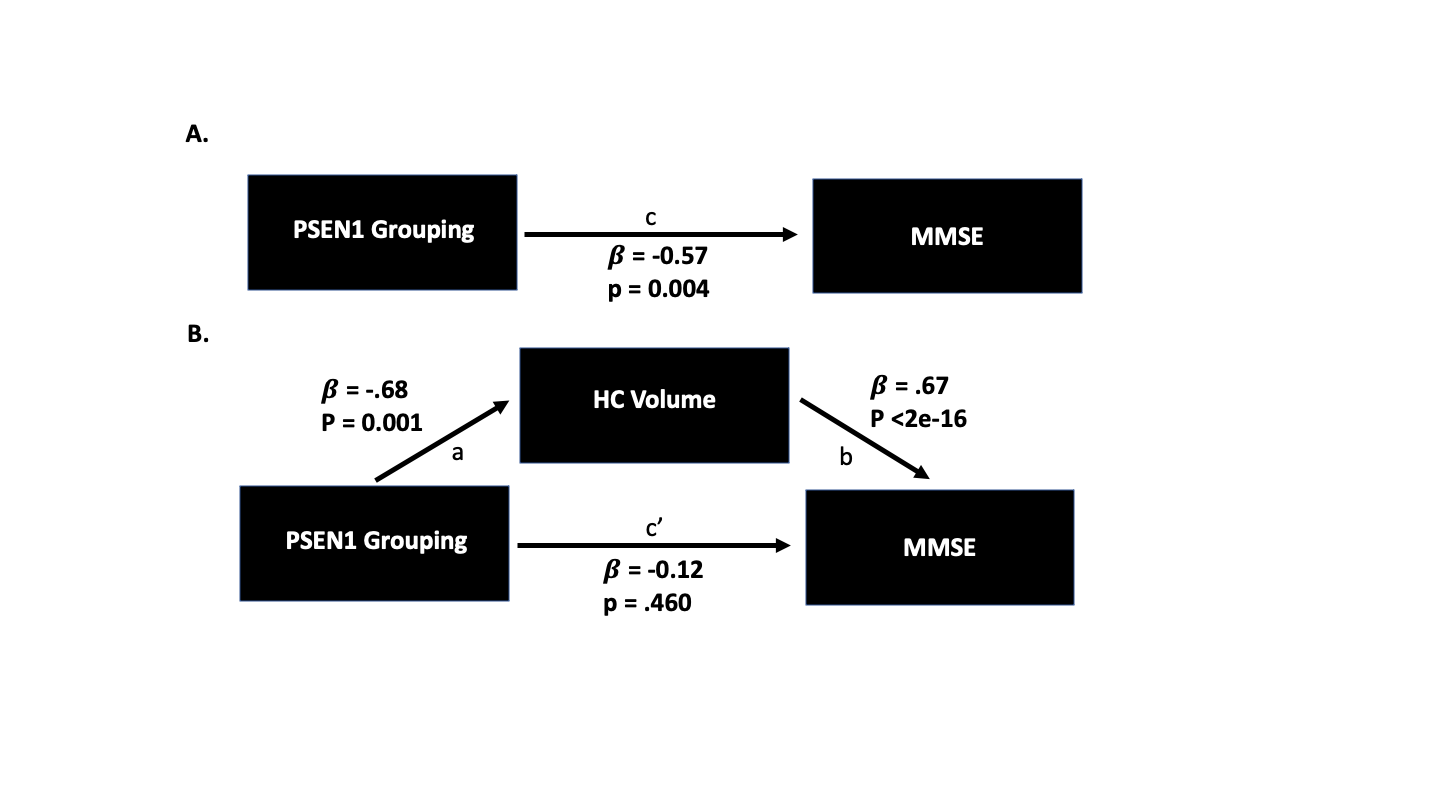
*

A mediation analysis was performed to examine whether the differences between PSEN1 CY vs TM groups on cognitive performance was mediated via hippocampal volume. This analysis was restricted to PSEN1 carriers who were at an EYO > -11.0, the point at which PSEN1 CY and TM groups diverge on gray-matter volumetrics. The significant association between PSEN1 grouping and MMSE (A) was no longer present when hippocampal volume was included as a mediator (B; residual direct effect c’ has p > 0.05), indicating a full statistical mediation.

**Figure S4.** *Similar levels of phospho-tau proteoforms are observed in CSF from PSEN1 CY and TM pathogenic variant carriers.*

**
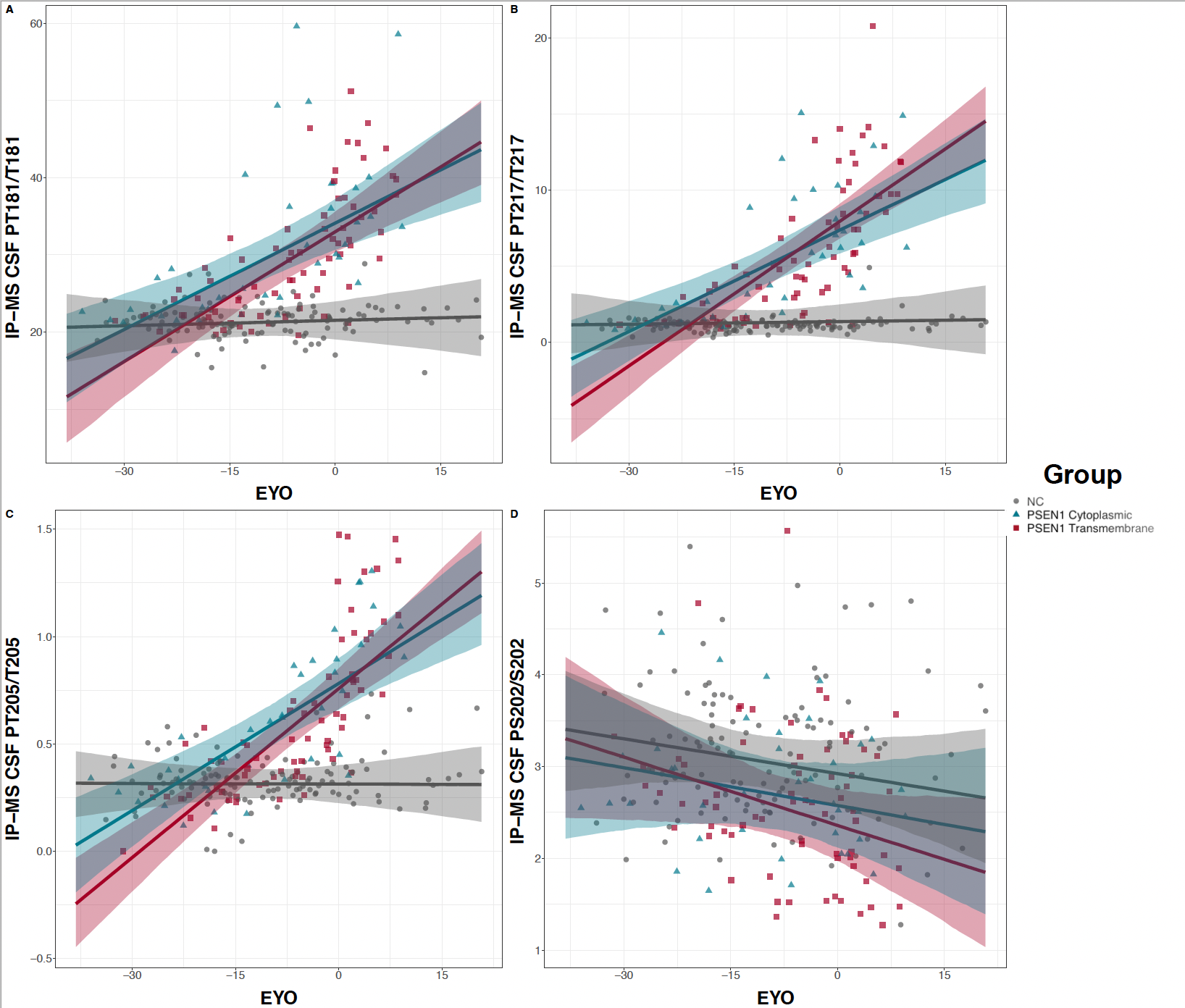
**

CSF IP-MS ratios of tau phosphorylation in pT181/ T181 (A), pT217/T217 (B), pT205/T205 (C), and pS202/S202 (D) are depicted for non-carriers (grey circles), CY carriers (blue triangles), and TM carriers (red squares), with respect to EYO. The shaded areas represent the 99% credible intervals around the model estimates derived by the Hamiltonian Markov chain Monte Carlo analyses. TM group did not differ from CY group across EYO on any of the CSF IP-MS phospho-tau isoform measures.

EYO = Expected years to symptom onset.

**Figure S5.** *Alternative variant grouping does not account for neurodegenerative, clinical, or cognitive heterogeneity in ADAD*


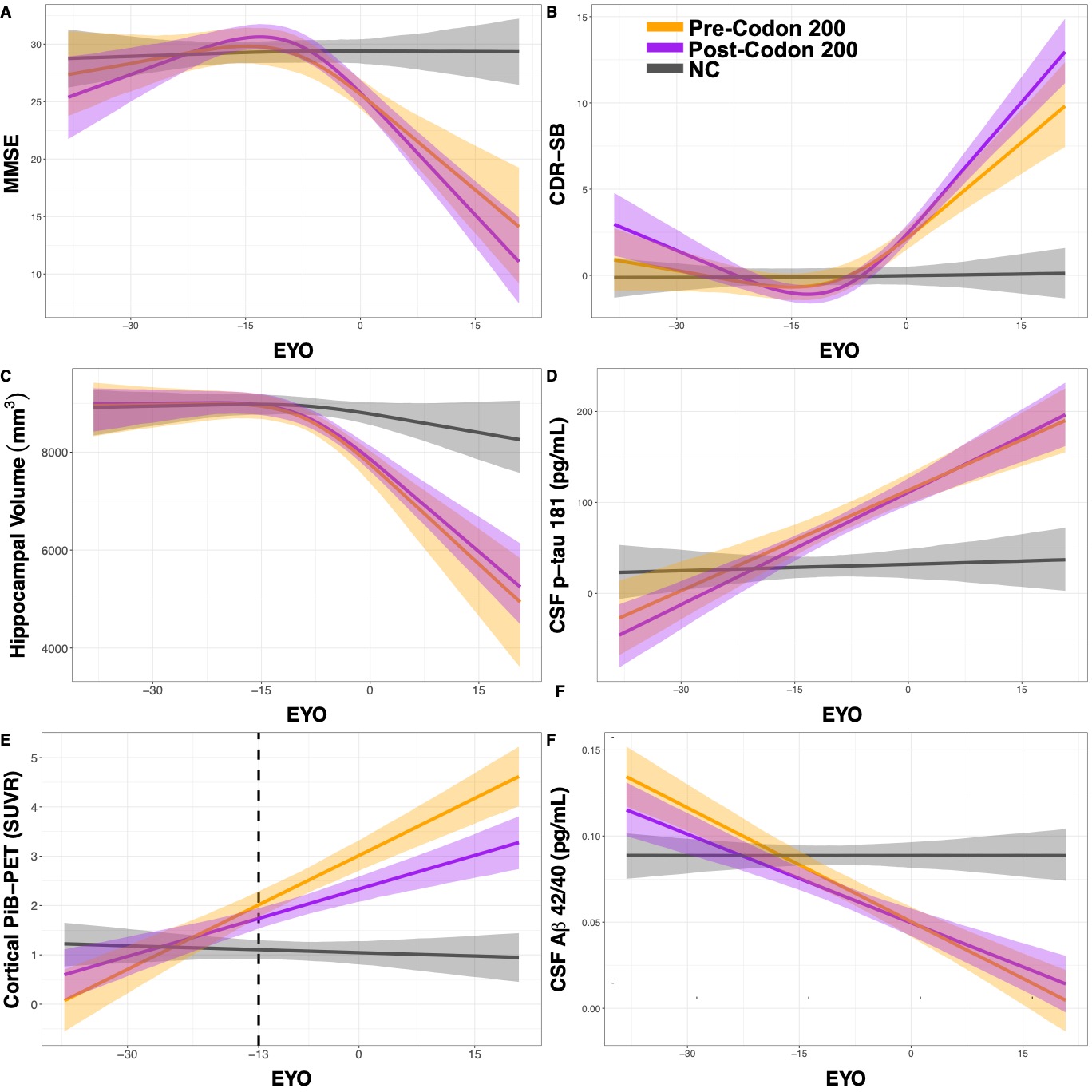


Cross-sectional A) Mini-Mental State Examination (MMSE), B) Clinical Dementia Rating-Sum of Boxes (CDR-SB), C) hippocampal volume (mm^3^), D) CSF phospho-tau 181 (pg/ml), E) Composite PiB-PET (SUVR), and F) CSF Aβ 42/40 (pg/mL) values for non-carriers (NC; grey), *PSEN1* pre-codon 200 pathogenic variant carriers (orange), and *PSEN1* post-codon 200 pathogenic variant carriers (purple), as compared to expected years to symptom onset (EYO). The solid line represents the median value of model estimates and the shaded areas represent the 99% credible intervals around the model estimates derived by the Hamiltonian Markov chain Monte Carlo analyses. The black dotted line in panel E indicates the EYO at which pre-codon 200 and post-codon 200 groups begin to diverge on cross-sectional measures of PiB-PET (EYO = -13). Note, a subset of data presented in panels B, E, F have been previously published in Chhatwal et al., 2022.
